# Identification of extracellular glycerophosphodiesterases in *Pseudomonas* and their role in soil organic phosphorus remineralisation

**DOI:** 10.1038/s41598-017-02327-6

**Published:** 2017-05-19

**Authors:** Ian D. E. A. Lidbury, Andrew R. J. Murphy, Tandra D. Fraser, Gary D. Bending, Alexandra M. E. Jones, Jonathan D. Moore, Andrew Goodall, Mark Tibbett, John P. Hammond, David J. Scanlan, Elizabeth M. H. Wellington

**Affiliations:** 1 School of Life Sciences, University of Warwick, Gibbet Hill Road, Coventry, West Midlands CV4 7AL United Kingdom; 2 The Earlham Institute, Norwich Research Park, Norwich, NR4 7UH United Kingdom; 3 School of Agriculture, Policy, and Development, University of Reading, Earley Gate, Whiteknights, Reading RG6 6AR United Kingdom; 4 Southern Cross Plant Science, Southern Cross University, Lismore, NSW 2480 Australia

## Abstract

In soils, phosphorus (P) exists in numerous organic and inorganic forms. However, plants can only acquire inorganic orthophosphate (Pi), meaning global crop production is frequently limited by P availability. To overcome this problem, rock phosphate fertilisers are heavily applied, often with negative environmental and socio-economic consequences. The organic P fraction of soil contains phospholipids that are rapidly degraded resulting in the release of bioavailable Pi. However, the mechanisms behind this process remain unknown. We identified and experimentally confirmed the function of two secreted glycerolphosphodiesterases, GlpQI and GlpQII, found in *Pseudomonas stutzeri* DSM4166 and *Pseudomonas fluorescens* SBW25, respectively. A series of co-cultivation experiments revealed that in these *Pseudomonas* strains, cleavage of glycerolphosphorylcholine and its breakdown product G3P occurs extracellularly allowing other bacteria to benefit from this metabolism. Analyses of metagenomic and metatranscriptomic datasets revealed that this trait is widespread among soil bacteria with *Actinobacteria* and *Proteobacteria*, specifically *Betaproteobacteria* and *Gammaproteobacteria*, the likely major players.

## Introduction

Although many forms of phosphorus (P) exist in soils, plants can only acquire simple inorganic orthophosphate (Pi) and therefore P scarcity often limits plant growth and hence crop production^1^. To alleviate P scarcity in soils, rock phosphate is heavily applied to many agricultural cropping systems. However, this application results in several negative environmental and geo-political consequences^2–4^. In addition to the secretion of organic acids that aid in the release of Pi chelated to various metal ions, plants also secrete an array of enzymes to facilitate the breakdown of various forms of complex P^5–7^. However, soil microorganisms also play a key role in both the solubilisation of Pi minerals and the remineralisation of organic forms of P and may help reduce our dependency on rock phosphate as a fertiliser^8, 9^. In soils, organic P often represents 30–65% of total P^10^, the majority of which are simple phosphomonoesters and phosphodiesters (up to 90%)^11^, or phytate (up to 50%)^9^. The organic P content of soil also contains phospholipids of both microbial and plant origin that are rapidly degraded resulting in the release of bioavailable Pi^12–14^. Furthermore, the addition of phospholipids to soil results in an increase in plant (Barley) P uptake^15^. However, the key genes and enzymes responsible for this potentially beneficial metabolic step have not been identified in soils.

Various microbial secreted enzymes (exoenzymes) including alkaline phosphatases (ALP), acid phosphatases (ACP), phytases, phosphonatases, nucleases and phosphodiesterases can facilitate the remineralisation of organic P into Pi thus improving soil P fertility^16–18^. These exoenzymes are usually secreted in response to Pi scarcity and this process, as well as the expression of other proteins, is regulated by a two component regulatory system, consisting of a DNA-binding transcriptional regulator (PhoB) and transmembrane histidine kinase (PhoR)^19^. In the rhizobacterium *Pseudomonas putida* BIRD-1, mutagenesis of the genes (*phoBR*) encoding PhoBR silenced the normal induction (upon Pi-depletion) of various Pi-scavenging proteins, as well as the high affinity Pi transporter, PstSABC^20^. This mutant could still grow on Pi due to the presence of a low affinity Pi transporter (PitA), whose expression was not affected by mutation of *phoBR* ^20^. ALPs, which are abundantly secreted in response to Pi-depletion^20, 21^, are thought to be promiscuous enzymes for numerous phosphomonoesters and phosphodiesters and their abundance and diversity is affected by bioavailable P and other environmental factors^22–26^.

Although ALPs are promiscuous enzymes, they cannot release Pi from glycerolphosphodiesters, such as glycerolphosphorylcholine (GPC), which forms the head group of phospholipids, such as phosphatidylcholine. In this instance, specific glycerolphosphodiester phosphodiesterases (GDPD, EC_3.1.4.46), either secreted (GlpQ) or located in the cytoplasm (UgpQ), are required to release *sn*-glycerol-3-phosphate (G3P) and the corresponding alcohol^27, 28^. Therefore, the release of Pi from glycerolphosphodiesters is a multi-step process with the final step performed by ALPs. GDPDs, namely GlpQ, produced by phylogenetically distinct bacteria such as *Escherichia*, *Bacillus*, *Streptococcus*, *Mycoplasma* and *Streptomyces* are often required for establishing various relationships (pathogenic or mutualistic) between bacteria and their recipient hosts^21, 27, 29, 30^. A periplasmic GlpQ was first characterised in *E*. *coli*, with the encoding gene (*glpQ*) co-located with *glpT*, the latter encoding a G3P transporter^27^. Interestingly, in *E*. *coli* the *ugpBAECQ* operon, which encodes a cytosolic GDPD and a corresponding ABC transporter for GPC, is induced by Pi depletion, whereas *glpQT* is part of the glycerol regulon^27, 28, 31, 32^. In contrast, in *B*. *subtilis* the *glpQT* operon is induced by Pi-depletion as well as by glycerol, but is repressed in the presence of a more favourable carbon source^21^. *Pseudomonas stutzeri* DSM4166, a nitrogen-fixing bacterium isolated from the rhizosphere of yellow indiangrass (*Sorghum nutans*)^33^, secretes numerous proteins in response to Pi-depletion^20^. The most abundant proteins found in the exoproteome of Pi-depleted *P*. *stutzeri* DSM4166 include ALPs, nucleotidases and a putative GDPD^20^. This putative GDPD contains the Pfam domain 03009, encompassing the GDPD family, found in both GlpQs produced by *E*. *coli* and *B*. *subtilis*. To date, the contribution of GlpQ to phospholipid transformations in soils is unknown.

Here, we determined whether the potential GlpQ from *P*. *stutzeri* DSM4166 is capable of converting GPC to G3P. We also identified and experimentally confirmed another GlpQ-type enzyme found in *P*. *fluorescens* SBW25. Furthermore, we also examined whether G3P was cleaved extracellularly releasing exogenous Pi. The consequence of this metabolism with respect to organic P cycling was investigated in a series of *Pseudomonas* co-cultivation experiments. Finally, we analysed *in silico* the distribution and transcription of GlpQ and other GDPD enzymes in various soil/rhizosphere systems. Together, our results present a mechanism for the release of Pi from phospholipid degradation in soil.

## Results

### Distribution of GlpQ-like homologs in *Pseudomonas* strains

Results from a previous exoproteomics experiment^20^ revealed that *P*. *stutzeri* DSM4166 secreted an exoprotein abundantly (encoded by PSTAA_4169) in response to low Pi availability. PSTAA_4169 is predicted to encode the GDPD domain (Pfam 03009) and showed homology to the periplasmic glycerolphosphodiesterase GlpQ found in *E*. *coli* (identity 36.78%, e-value, 2.0 e-54). PSTAA_4169, hereafter termed *glpQI*, encodes a protein (GlpQI) containing all the conserved residues associated with characterised GDPDs^29^ (Fig. S1). Thus, it was hypothesised that this Pi-responsive exoprotein is capable of cleaving the diester (C-O-P-O) bond associated with the headgroup of phospholipids. GlpQI was 50 amino acids longer than GlpQ homologs from other *P*. *stutzeri* strains and the cleavage site for the signal peptide of this protein is predicted to be located between amino acid residues 78–79. Furthermore, no peptides before the predicted signal P region were detected in the exoproteome of *P*. *stutzeri* DSM4166^20^, suggesting that GlpQI is mis-annotated in this strain.

**Figure 1:**
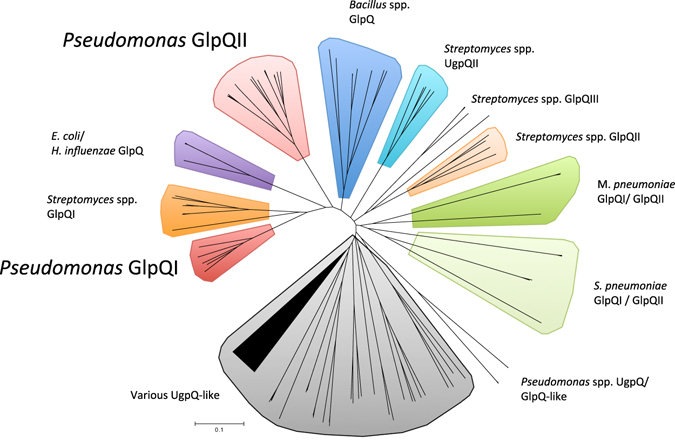
Phyologenetic analysis of GlpQI (DSM4166) and GlpQII (SBW25) in relation to other characterised and non-characterised proteins containing the Pfam domain – 03009. A full list of genomes used in the search is provided in Table S5. The evolutionary history was inferred using the Neighbor-Joining method^50^. The optimal tree with a sum of branch length = 17.19752950 is shown. The tree is drawn to scale, with branch lengths in the same units as those of the evolutionary distances used to infer the phylogenetic tree. The evolutionary distances were computed using the p-distance method and are in the units of the number of amino acid differences per site. The analysis involved 144 amino acid sequences. All ambiguous positions were removed for each sequence pair. There were a total of 483 positions in the final dataset. Evolutionary analyses were conducted in MEGA6^51^. Bootstrap values were omitted for clarity.

To better understand the phylogeny of GDPD-like proteins, a number of *Pseudomonas* strains as well as various phylogenetically distinct bacteria (e.g. *Mycoplasma pneumoniae*, *Streptococcus pneumoniae*, *Haemophilus influenzae*), whose genomes have been deposited in the Integrated Microbial Genomes database at the Joint Genome Institute (IMG/JGI) and are known to harbour genes encoding previously characterised GlpQ homologs (Table S5), were screened for the presence of genes encoding enzymes containing the Pfam 03009 domain. The characterised UgpQ from *E*. *coli* ^34^ and other cytoplasmic GDPDs (putative and characterised) were retrieved using this method. The various GDPDs clustered into several groups, including one group that possessed homologs of GlpQI (Fig. 1). In this group, homologs were predominantly retrieved from various *P*. *stutzeri* strains with no homologs of GlpQI found in the majority of other well-known *Pseudomonas* species e.g. *P*. *putida*, *P*. *fluorescens*, or *P*. *syringae*. However, we also identified another cluster (termed GlpQII in Fig. 1) that contained several sequences also predicted to possess signal peptides. One of these sequences, retrieved from *P*. *fluorescens* SBW25 (ORF, PFLU4789, hereafter referred to as GlpQII), showed similarity to GlpQI (Identity 28.77%, e-value 4.0 e-23). The GlpQII cluster also contained sequences retrieved from other *Pseudomonas* spp. Like GlpQI produced by *P*. *stutzeri*, GlpQII also contained the conserved residues required for GDPD activity. Interestingly, the genomes of *P*. *aeruginosa* strains encode the genes for both GlpQI and GlpQII.

### GlpQ is essential for growth on glycerolphosphodiesters in *Pseudomonas*

**Figure 2:**
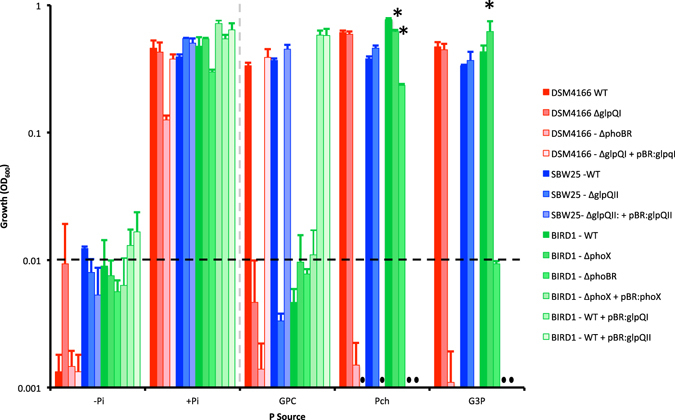
Growth of three *Pseudomonas* strains on differing organic phosphorus (P) compounds as a sole P source (100 μM) using succinate (20 mM) as the carbon source. Results shown are the mean of triplicate cultures. The dashed black line indicates approximate OD_600_ at T = 0. The dashed grey line segregates the positive and negative controls to the organic P treatments. Asterisks denote a significant (T-test, P < 0.05) reduction in growth rate (Fig. S5). Error bars denote standard deviation. Abbreviations: −P, no added orthophosphate; +P 100 µM orthophosphate added; Pch, phophorylcholine; G3P, *sn*-glycerol-3-phosphate; GPC, glycerolphosphorylcholine; DSM4166, *P*. *stutzeri* DSM4166; SBW25, *P*. *fluorescens* SBW25; BIRD-1, *P*. *putida* BIRD-1; WT, wild type; *ΔglpQ*, mutant genotype for the secreted glycerolphosphodiesterase; *ΔphoBR*, mutant genotype for the master regulator of the phosphate starvation response network; *ΔphoX*, mutant genotype for alkaline phosphatase.

To test the hypothesis that these *Pseudomonas* GlpQ-like homologs have a role in growth on phospholipid headgroups, we cultured *P*. *stutzeri* DSM4166 and *P*. *fluorescens* SBW25 with GPC as the sole source of P (100 μM) (Fig. 2). We also grew *P*. *putida* BIRD-1 on GPC as the sole source of P as this bacterium does not encode GlpQI or GlpQII in its genome. The wild type (WT) strains of *P*. *stutzeri* DSM4166 and *P*. *fluorescens* SBW25 grew on the phosphodiester GPC whilst *P*. *putida* BIRD-1 did not, suggesting that GlpQI and GlpQII can function as a GDPD (Fig. 2). All three WT strains grew on G3P, the product of characterised GlpQ-mediated GPC catabolism, and phosphorylcholine (Pch) (Table 1).

**Table 1:** Growth characteristics of the various strains and mutants used in this study with respect to P utilisation.

Strain	Genotype	Low Pi	High Pi	Pch	G3P	GPC
*P*. *stutzeri* DSM4166	Wild type	**+**	**+**	**+**	**+**	**+**
	*ΔglpQII* − disrupted *glpQI*	**+**	**+**	**+**	**+**	X
	*ΔphoBR* − silenced pho regulon, inc. *pstSABC*	**+** ^1^	**+**	X	X	X
	*ΔglpQI* + *pBR*:*glpQI* − complemented with native *glpQI*	**+**	**+**	**+**	**+**	**+**
*P*. *fluorescens* SBW25	wild type	**+**	**+**	**+**	**+**	**+**
	*ΔglpQII* − *disrupted glpQII*	**+**	**+**	**+**	**+**	X
	*ΔglpQII* + *pBR*:*glpQII* − complemented with native *glpQII*	**+**	**+**	**+**	**+**	**+**
*P*. *putida* BIRD-1	wild type	**+**	**+**	**+**	**+**	X
	+*pBR*:*glpQI* − heterologously expressing *glpQI*	**+**	**+**	**+**	**+**	**+**
	+*pBR*:*glpQII* − heterologously expressing *glpQII*	**+**	**+**	**+**	**+**	**+**
	*ΔphoBR* − silenced pho regulon, inc. *pstSABC*	**+** ^1^	**+**	**+** ^1^	**+** ^1^	X
	*ΔphoX* − disrupted ALP	**+**	**+**	**+**	**+** ^1^	X

Abbreviations: Pi, orthophosphate; Pch, phosphorycholine; G3P, glycerol 3-phosphate; GPC, sn-glycerol phosphorylcholine; ALP, alkaline phosphatase; *pstSABC*, genes encoding the high-affinity Pi transcporter.

^1^Growth occurred, but the rate was slower than that of the wild type.

In addition to *glpQI* and *glpQII*, *P*. *stutzeri* DSM4166 and *P*. *fluorescens* SBW25 also possess *plcP*, which encodes an intracellular phospholipase C^20, 34–36^, and another gene encoding the Pfam03009 domain (PSTAA_2726, PFLU1570). Therefore, to further investigate if GlpQI and GlpQII were responsible for growth on exogenous GPC in both *P*. *stutzeri* DSM4166 and *P*. *fluorescens* SBW25, respectively, a *ΔglpQ* mutant was constructed for each strain by marker exchange mutagenesis. Both *ΔglpQ* mutant variants of *P*. *stutzeri* DSM4166 and *P*. *fluorescens* SBW25 no longer grew on GPC as a sole source of P. However, both mutant strains could still utilise G3P and Pch (Fig. 2). Plasmid-encoded complementation of either mutant with their respective native *glpQ* restored growth on GPC (Fig. 2). Furthermore, we also separately introduced the two plasmids harbouring either *glpQI* or *glpQII* into the wild type strain of *P*. *putida* BIRD-1. Complementation with either *glpQ* gene conferred the ability of this bacterium to utilise GPC as a sole source of P. Together, these data confirm that *glpQI* and *glpQII* encode two phosphodiesterases capable of cleaving phospholipid headgroups.

### *Pseudomonas* strains perform all the catabolic steps of GPC metabolism extracellularly

No apparent transporter for the predicted metabolite of GPC cleavage (glycerol 3-phosphate, G3P), namely GlpT nor UgpABCE, is present in the genome of *P*. *stutzeri* DSM4166. Therefore we hypothesised that G3P degradation occurs extracellularly via one of its ALPs (PhoXI, PhoXII and PhoD) that are also abundantly secreted in response to Pi-depletion^20^. To test this hypothesis, a mutant targeting the two component master regulator of the Pi-stress response, *ΔphoBR*, was constructed in an attempt to silence all three ALPs. As expected, the *ΔphoBR* mutant of *P*. *stutzeri* DSM4166 no longer expressed any inducible APase activity when grown under Pi-deplete conditions indicating that all three ALPs were indeed silenced (Fig. S2). Moreover, this strain no longer grew on either GPC, or its metabolites G3P or PhC (Fig. 2). Similarly, a *ΔphoBR* mutant of *P*. *putida* BIRD-1, which also lacks APase activity^20^, also failed to grow on G3P as a sole source of P (Fig. 2). However, in contrast to the *P*. *stutzeri* DSM4166 *ΔphoBR* mutant, the BIRD *ΔphoBR* mutant still grew slowly on Pch (Fig. 2), indicating that a PhoBR-independent enzyme can still catabolise this compound. Indeed, the genome of *P*. *putida* BIRD-1 contains several ORFs that encode putative phosphatases^37^. Unlike *P*. *stutzeri* DSM4166, which has three APases, *P*. *putida* BIRD-1 only possesses and secretes one ALP (PhoX) in response to Pi-depletion^20^. Therefore a *ΔphoX* mutant of *P*. *putida* BIRD-1 was constructed. The *ΔphoX* mutant also lacked any inducible ALP activity (Fig. S3) and its growth rate when grown on G3P as a sole P source was severely affected (Fig. S4), although it did finally reach similar final cell yields (Fig. 2), demonstrating that PhoX has a role in G3P metabolism. Even so, the fact that the *ΔphoX* mutant grew slowly on G3P whilst the *ΔphoBR* mutant did not suggests that *P*. *putida* BIRD-1 does produce a PhoBR-dependent enzyme capable of degrading G3P. Furthermore, complementation of the *ΔphoX* mutant with the native *phoX* restored phosphatase activity (Fig. S3). Together, these data suggest that *Pseudomonas* cleaves GPC and its metabolites extracellularly, utilising the high affinity phosphate transport (Pst) system to take up the newly released Pi into the cell.

### GPC-utilising bacteria can liberate P for the bacterial community

**Figure 3:**
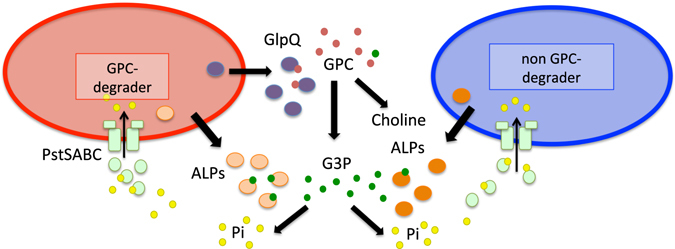
A proposed model for the metabolic steps of glycerolphosphorylcholine (GPC) degradation among *Pseudomonas* strains. Each cleavage step occurs either in the periplasm or extracellularly resulting in the production of free inorganic orthophosphate (Pi). Cells can then take up the Pi through Pi-specific transporters. Abbreviations: ALPs, alkaline phosphatases; PstSABC, high-affinity Pi transporter; other abbreviations are as stated in the previous figure.

**Figure 4:**
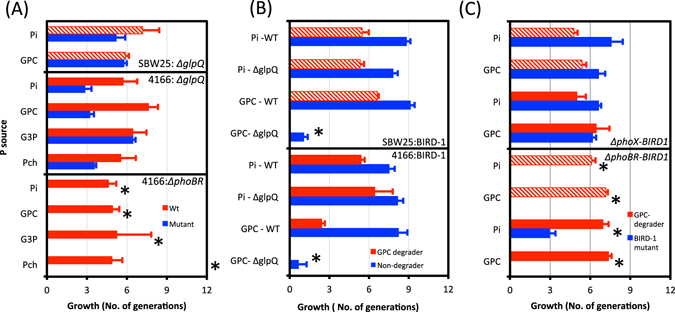
Co-cultivation experiments using various *Pseudomonas* strains assessing growth on extracellular glycerolphosphorylcholine (GPC) (100 μM), the potential GPC cleavage product glycerol 3-phosphate (G3P), phosphocholine (Pch), as well as growth on orthophosphate (Pi). Either *P*. *stutzeri* DSM4166 (block red) or *P*. *fluorescens* SBW25 (hashed red) were used as the GPC utiliser. All blue bars indicate when a strain was the proposed beneficiary of GPC catabolism. Growth was determined by calculating the number of generations based on plate counts for each strain in the co-cultivation experiment. Asterisks represent conditions where the non-GPC degrader did not grow or when growth of a given mutant was significantly reduced compared to the wild type (T-Test P < 0.05). (**A**) Growth of the *ΔglpQ* mutant in the presence of the parental wild type (WT) strain revealed that the mutants could still grow despite lacking the ability to degrade GPC (Upper and Middle Panel). Note that the *P*. *stutzeri* DSM4166 *ΔphoBR* mutant (lower panel) showed a severe fitness defect (due to loss of expression of the high affinity Pi transporter). (**B**) Both the wild type and *ΔglpQ* mutants for *P*. *stutzeri* DSM4166 and *P*. *fluorescens* SBW25 were co-cultivated with *P*. *putida* BIRD-1 on either Pi or GPC. (**C**) *P*. *stutzeri* DSM4166 and *P*. *fluorescens* SBW25 were co-cultivated with various *P*. *putida* BIRD-1 mutants (*ΔphoX* and *ΔphoBR*). Again, the BIRD-1 *ΔphoBR* mutant does not express the high-affinity Pi transporter. Results shown are the mean of triplicate cultures. Error bars denote standard deviation.

Based on these results, we propose a model for GPC degradation illustrating how non-GPC degrading bacteria may benefit from its extracellular breakdown (Fig. 3). To test this hypothesis a series of co-cultivation experiments were established with different combinations of various *Pseudomonas* strains. Initially, the *P*. *stutzeri* DSM4166 or *P*. *fluorescens* SBW25 wild type were co-cultured with their respective *ΔglpQ* mutant strains with low Pi (100 μM) or GPC (100 μM) as the sole P source. Although neither *ΔglpQ* mutant could grow on GPC in isolation (Fig. 2), the mutants did grow in the presence of their parental wild type strains (Fig. 4A). *P*. *fluorescens* SBW25 does possess a *glpT* homolog (identity 74.31%, e-value 0.0e + 00) to *E*. *coli glpT*), therefore it was interesting that the corresponding *ΔglpQ* mutant could still utilise the G3P produced by GlpQ. In addition, when *P*. *putida* BIRD-1 (non-GPC utilising) was co-cultivated with wild type *P*. *stutzeri* DSM4166 or *P*. *fluorescens* SBW25 (GPC-utilising) using GPC as the sole P source, *P*. *putida* BIRD-1 grew well (Fig. 4B). However, when *P*. *putida* BIRD-1 was co-cultivated with either *ΔglpQ* mutant, no growth was observed. These results indicate that once GPC is cleaved to G3P, other non GPC-utilising strains can compete for this cleavage product.

Thus far, our data revealed that secreted APases are responsible for the catabolism of G3P, the breakdown product of GPC. To confirm that the extracellular cleavage of G3P could cross-feed Pi into another bacterium we co-cultivated the *ΔphoX* mutant of *P*. *putida* BIRD-1 with its parental WT strain. When grown on G3P or Pch the *ΔphoX* mutant showed no reduction in fitness (Fig. S5). However, replicating the same experiments with the *ΔphoBR* mutant revealed a severe fitness reduction to the mutant (Fig. S5). This approach was taken a step further by co-cultivating either the *P*. *putida* BIRD-1 *ΔphoBR* or *ΔphoX* mutants with the parental wild type strains of *P*. *fluorescens* SBW25 or *P*. *stutzeri* DSM4166 using GPC as the sole source of P (Fig. 4C). As expected, the *ΔphoX* mutant showed no fitness reduction when co-cultivated with either SBW25 or *P*. *stutzeri* DSM4166 under low Pi (100 μM) or GPC indicating that it could successfully compete for the Pi that was released by GlpQ and ALPs. In contrast, the *P*. *putida* BIRD-1 *ΔphoBR* mutant had a significantly reduced ability to compete against either *P*. *fluorescens* SBW25 or *P*. *stutzeri* DSM4166 when either low Pi or GPC was the sole source of P (Fig. 4C). A similar result was also observed when both the *P*. *stutzeri* DSM4166 *ΔphoBR* mutant and its parental wild type strain were co-cultivated together and grown on GPC as the sole P source (Fig. 4A, lower panel). We observed no fitness defect in the *ΔphoBR* mutants when co-cultivated at high Pi concentrations (Fig. S6), presumably because low affinity Pi transporters (namely PitA; PFLU1359, PFLU4794; PPUBIRD1_1747; PPUBIRD1_4189; PSTAA_0323) are the main routes for extracellular Pi uptake under these conditions. Together, these results demonstrate that 1) the catabolism of phospholipid headgroups occurs outside the cell through the utilisation of extracellular enzymes and 2) PstSABC, expressed under concentrations of low exogenous Pi^20^, is utilised to competitively take up the liberated Pi.

### Distribution of GlpI and GlpII in soil metagenomes

**Figure 5:**
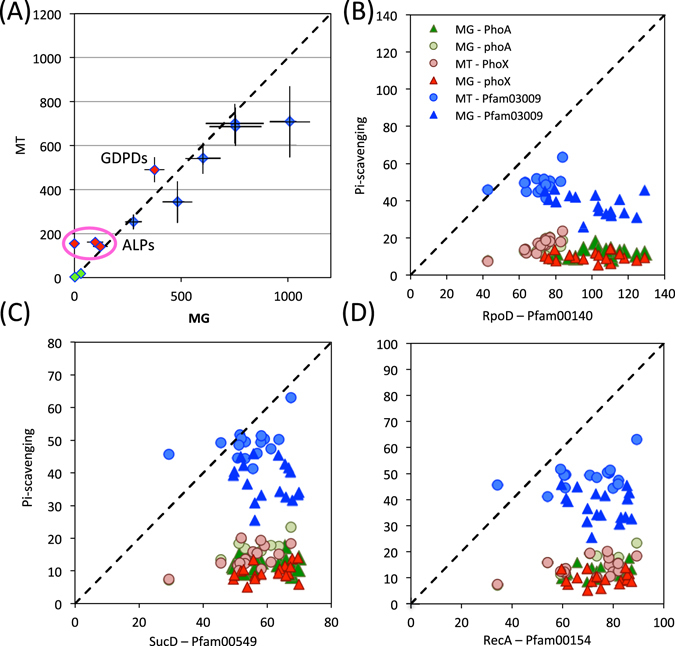
The abundance of various genes and their corresponding transcripts encoding Pfam domains associated with various phosphate (Pi)-scavenging (pink diamonds), nitrogen fixing (green diamonds) and housekeeping functions (blue diamonds) sampled at the Centre INRS-Institut Armand-Frappier, Laval, Canada (**A**). The mean (n = 16) number of reads assigned to each Pfam domain retrieved from either the metagenomes (MG) or metatranscriptomes (MT). Error bars denote standard deviation. The number of reads assigned to various Pfam domains associated with Pi-scavenging were plotted against the number of reads assigned to Pfam domains associated with RpoD (**B**), SucD (**C**), RecA (**D**). Abbreviations: ALPs, alkaline phosphatases; GDPDs, glycerolphosphodiesterase).

To gain a better understanding of the abundance and subsequent expression of GDPDs in agricultural soils, we screened a number of metagenomes (MG) and metatranscriptomes (MT) generated from the Centre INRS-Institut Armand-Frappier, Laval, Canada, deposited in the IMG/JGI database (Project ID – Gp0115425). Functional searches were performed using certain Pfam domains associated with Pi-scavenging enzymes: Pfam03009 (GDPD), Pfam05787 (PhoX), Pfam00245 (PhoA), as well as various Pfam domains (Pfam00154/10415/00549/00140) associated with genes encoding housekeeping proteins (RecA/SucD/RpoD) as the queries. In order to compare Pi-scavenging function with another important soil function, Pfam domains associated with genes encoding the nitrogen fixation enzymes NifH and NifQ were also added to the analysis. As expected, in both the MGs and MTs a number of reads were assigned to Pfam domains associated with housekeeping functions. Interestingly, we also found a number of reads associated with ALPs (PhoX, PhoA, PhoD) and GDPDs in both the MGs and MTs whilst only a few reads that were assigned to Pfam domains associated with NifH/Q were retrieved (Fig. 5A). The number of reads associated with GDPDs and ALPs (PhoX, PhoA, PhoD) was slightly greater in the MTs compared to the MGs (Fig. 5A). Indeed, the ratio of reads associated with Pi-liberating functions to housekeeping domains (RecA, RpoD, SucD) was greater in MTs than it was in MGs (Fig. 5B–D) confirming genes related to Pi scavenging were being transcribed and presumably expressed in these soil and rhizosphere samples.

As Pfam03009 is a general property of GDPDs, this analysis likely retrieved hits related to intracellular GDPDs, such as UgpQ. To better determine the number of GlpQ-like enzymes in these environments, BLASTP (cut off = e-20, 40% identity) searches using GlpQI and GlpQII from *Pseudomonas*, as well as GlpQ from *Bacillus* and *Escherichia* were also performed on each dataset. Using GlpQI retrieved 430 hits whilst GlpQII only returned 16. GlpQ from *Bacillus* and *Escherichia* returned 34 and 23 hits, respectively. Combining all search results together resulted in a collection of 462 distinct hits (Fig. S6). Therefore, BLASTP searches returned an order of magnitude less hits than searching using the Pfam domain 03009.

The two major GlpQ producers in these soil environments are *Actinobacteria* and *Proteobacteria*, whist a number of hits were also assigned to *Firmicutes*, *Gemmatiomonadetes*, *Cyanobacteria* and *Acidobacteria* (Fig. S7A). In MGs the ratio between reads assigned to *Proteobacteria* and *Actinobacteria* was 2:1. However, in the MTs this became a 1:1 ratio (Fig. S7A), suggesting that both of these phyla play equally important roles in glycerolphosphodiester degradation in the soil. Based on their relative abundance in the MTs, the *Gamma*- and *Betaproteobacteria* appeared to be the two major classes responsible for glycerolphosphodiester degradation in soils.

**Figure 6:**
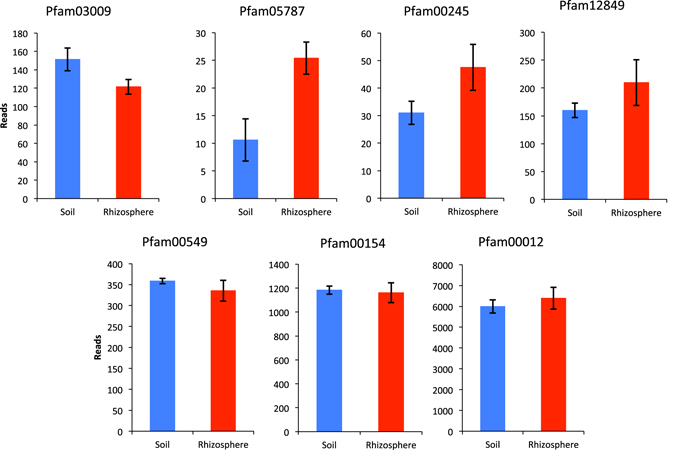
The number of transcripts encoding Pfam domains associated with various phosphate (Pi)-scavenging and housekeeping functions detected in the metatranscriptomes (MT) sample at the Kellogg Biological Station, Michigan, USA. Results shown are the mean of triplicate cultures. Error bars denote standard deviation. Pfam03009, glycerolphosphodiester phosphodieasterase; Pfam05787, alkaline phosphatase – PhoX; Pfam00245, alkaline phosphatase – PhoA; Pfam16655, alkaline phosphatase – PhoD; Pfam12849, high-affinity Pi substrate binding protein– PstS; Pfam00549, SucD; Pfam00154, RecA; Pfam00012, DnaK.

Finally, in order to better understand the cycling of phospholipid headgroups in the rhizosphere, we compared bulk soil versus rhizosphere MT samples from the Kellogg Biological Station, Michigan, USA, again using the function search option in IMG/JGI. The number of transcripts assigned to Pfam domains associated with housekeeping genes was comparable (unpaired T-Test, P > 0.05) between soil and rhizosphere samples (Fig. 6). There were a greater number of transcripts assigned to PhoA, PhoX and PstS in the rhizosphere, although only PhoX was statistically significant (unpaired T-test = P < 0.05). However, there was a statistically significant (unpaired T-test = P < 0.05) reduction in the number of transcripts assigned to GDPDs in the rhizosphere compared to bulk soil. This result was also confirmed by BLASTP (cut-off - e-20, 30% identity) analysis using GlpQI from *P*. *stutzeri* DSM4166 as the query, where 20 hits were retrieved from bulk soil and only 4 were retrieved from the rhizosphere samples.

Taken together, these data reveal that genes involved in liberating Pi from glycerolphospholipid headgroups are abundant, derived from a diverse range of bacterial taxa and are expressed in the soil.

## Discussion

Whilst non-specific ALP and phosphodiesterase activities (and to a lesser extent phosphonate and phytate degradation) have been extensively surveyed in various soils^22–26, 38^, the activity of GDPDs in soils has received little attention. Using a combination of bioinformatic and genetic approaches we identified and characterised two secreted GDPDs in soil-dwelling/plant associated *Pseudomonas* species essential for growth on exogenous GPC, termed GlpQI (from *P*. *stutzeri* DSM4166) and GlpQII (from *P*. *fluorescens* SW25). Whilst the degradation of phospholipids and their corresponding headgroups in soil is poorly documented, limited evidence suggests that their degradation results in an increase in soil Pi^14^. GlpQI and ALPs are among the most abundant exoenzymes detected in the exoproteomes of Pi-depleted *Pseudomonas* and *Bacillus* cells^20, 21^ suggesting that these enzymes are likely responsible for this release of Pi from phospholipids. Here, as well as providing genetic evidence for the function of two distinct *Pseudomonas* GlpQs (Fig. 2), we also performed a series of co-culture experiments to provide ‘proof-of-concept’ that phospholipid headgroups are degraded extracellularly and that non-GPC utilising bacteria can benefit from the action of secreted GlpQ (Fig. 4). The fact that only the *Pseudomonas ΔphoBR* mutants, unable to express the PstSABC transporter^20^, could no longer grow in co-cultivation with a GPC-degrader strengthens the hypothesis that Pi is released extracellularly (lower sections of Fig. 4A,C). In *E*. *coli* GlpQ is thought to be localised to the periplasm^27^, so whether *Pseudomonas* GlpQ enzymes are primarily located in the periplasm & leak outside the cell non-specifically as seen for other periplasmic proteins^20, 39, 40^ or are truly ‘secreted’ proteins, remains to be determined. GlpQ was also an abundant protein in the *Bacillus* exoproteome during Pi depletion suggesting that this enzyme may be secreted^21^.

*P*. *fluorescens* SBW25 was isolated from the phytosphere of sugarbeet and is an effective coloniser of the pea rhizosphere^41^, whereas *P*. *stutzeri* DSM4166 was isolated from the rhizosphere of yellow indiangrass (*Sorghum nutans*)^33^. Like other GlpQ-harbouring bacteria, both of these *Pseudomonas* strains form close associations with eukaryotic organisms suggesting that the ability to degrade phospholipid headgroups confers an ecological advantage in these environments^27, 29, 30, 37^. Interestingly, in *P*. *stutzeri* DSM4166, growth on GPC as a sole P source was also dependent on a functional *pho* regulatory system as the *phoBR* knockout mutant failed to grow (Fig. 2). This observation, coupled with its abundant secretion under Pi depletion^17^ suggests that GPC may be utilised primarily as a source of P in the rhizosphere which is often a limiting substrate for bacteria whose primary nutrient source is carbon-rich plant root exudates, namely amino acids, organic acids and carbohydrates^42^.

Whilst GlpQI in *P*. *stutzeri* DSM4166 was abundantly secreted in response to Pi-depletion, GlpQII in *P*. *fluorescens* SBW25 was not among the most abundant exoproteins found in its exoproteome, but was still responsive to Pi depletion^17^. This observation may be attributed to differences in regulatory capacity in these two strains. However, both *glpQI* and *glpQII* are found in the genome of *P*. *aeruginosa* and in this bacterium *glpQI* showed a greater up-regulation (25-fold) compared to *glpQII* (5.8-fold) in response to Pi-depletion^43^. A similar regulatory pattern was observed in *Mycoplasma pneumoniae*, with the suggestion that the two GlpQ enzymes may have differing substrate specificities and physiological roles within the cell^36^. Although not investigated in this study, expression of GlpQs is induced/repressed by carbon sources and carbon limitation^16, 28, 38^, which may also affect the levels of Pi released from phospholipids in soils.

Whilst recent studies have addressed the distribution of ALPs in soil^22–25^, the distribution of GDPDs, including GlpQ, in this environment is unknown. Although phospholipids are ubiquitous in bulk soil^10^, we hypothesised that their abundance may be higher in the rhizosphere due to plants releasing mucilage that is partly composed of phosphatidylcholine^9^. Furthermore, the rhizosphere is often Pi-depleted which may lead to induction of *glpQ* akin to the induction of ALP activity^21, 22, 33^. Subsequently, we predicted that an enrichment of the genes encoding GDPDs, and their corresponding transcripts, may be higher in the rhizosphere compared to bulk soil. However, whilst we found no evidence that genes encoding GDPDs are differentially transcribed in the rhizosphere and bulk soil, the number of transcripts assigned to ALPs (PhoX and PhoA) was higher in the rhizosphere (Fig. 6). Indeed, phosphatase activity (at pH 6.5) was higher in *P*. *putida* BIRD-1 cells retrieved from the rhizosphere compared to cells retrieved from the bulk soil^37^ indicating an induction of PhoX or similar exoenzymes. Interestingly, glycerolphosphorylethanolamine, which is generally the major head group of phospholipids found in bacteria^12^, as well as other glycerolphosphodiesters were also substrates for *E*. *coli* GlpQ^27^. This may help explain the abundance of *glpQ* in bulk soil samples. Therefore, investigating the substrate specificity of various soil-dwelling GlpQ enzymes warrants future attention.

It appears that multiple phyla are contributors to phospholipid head group degradation. The genomes of *Actinobacteria* often contain multiple forms of *glpQ* ^44^ whilst *Proteobacteria*, generally contain only one copy of *glpQ* and a putative *ugpQ*. *Betaproteobacteria*, which are frequently enriched in the rhizosphere of plants^45, 46^, also appear to be important recyclers of glycerolphosphodiesters, as both MGs and MTs had a number of hits related to this class (Fig. S7). Inspection of genomes deposited in the National Centre for Biotechnology information (NCBI) database related to the *Betaproteobacteria* did indeed reveal this class does possess a number of genes encoding secreted GDPDs, with homology to GlpQ (Table S3).

In conclusion, the current study provides evidence for a mechanism to explain the observed Pi release from phospholipid degradation in soils. Using *Pseudomonas* as a model, our data demonstrates how the extracellular cleavage of GPC and its breakdown products can cross-feed P into the wider microbial community and likely help plants acquire more Pi. Finally, the capacity for this potential plant-growth promoting effect is not confined to *Pseudomonas*, but is present in various phylogenetically distinct soil bacteria.

## Materials and Methods

### Bacterial strains and growth conditions

*Pseudomonas putida* BIRD-1, *P*. *fluorescens* SBW25, and *P*. *stutzeri* DSM4166 were maintained on Luria Bertani (LB) agar (1.5% w/v) medium at 30 °C. Their respective mutants were maintained on similar plates containing the appropriate antibiotic. For all growth experiments triplicate cultures were grown in an adapted Minimal A medium comprising: Na-Succinate 5.4 g L^−1^, NaCl 200 mg L^−1^, NH_4_Cl 450 mg L^−1^, CaCl_2_ 200 mg L^−1^, KCL mg L^−1^ MgCl_2_ 450 mg L^−1^, FeCl_2_ 10 mg L^−1^, MnCl_2_ 10 mg L^−1^, 10 mM 4-(2-hydroxyethyl)-1-piperazineethanesulfonic acid (HEPES) pH 7.2. KH_2_PO_4_, Pch, G3P, or GPC were added to a final concentration of either 100 μM or 200 μM. *Pseudomonas* species were pre-cultured in minimal A medium containing 100–200 μM Pi to ensure cells had adequate Pi for growth whilst minimising the potential for carry over of residual Pi into triplicate experimental cultures.

### Co-cultivation experiments with various *Pseudomonas* strains

Starter cultures for each species contained minimal A medium supplemented with 100 μM Pi and incubated overnight. Each strain was inoculated (2%) in an approximate 1:1 ratio (n = 3). The number of colony forming units (CFU) were determined by plate counting (n = 3) at T0 and again (T1) after overnight incubation at 30 °C. LB agar was used to determine overall bacterial counts and additions of gentamicin or chloramphenicol were used as appropriate to select for mutants and or *P*. *putida* BIRD-1 wild type. Where no antibiotics could be used, colony morphology was used to distinguish organisms (DSM4166: BIRD-1). The number of generations was determined by calculating the change in CFUs between T0 and T1.

### Generation of the various *Pseudomonas* mutants

To construct the various *Pseudomonas* mutants, we used the methods developed by Lidbury *et al*.^20^ that were previously employed to construct the *P*. *putida* BIRD-1 *ΔphoBR* mutant. A full list of strains, plasmids and primers used in this study is outlined in Tables S2 and S3. Initially, two regions of genomic DNA, one at the 5′ end and the other at the 3′ end of the given functional gene were PCR amplified, along with the gentamicin resistance cassette from p34S-Gm^47^. The suicide vector pK18mob*sacB* ^48^ was linearised using the restriction enzymes *Bam*HI and *Hin*dIII and all four fragments of DNA were ligated together using the HiFi DNA Assembly Kit (New England Biolabs, Hitchin, UK) according to the manufacturer’s guidelines. The resulting plasmid was transformed into *Escherichia coli* S17.1 via electroporation and mobilized into the respective *Pseudomonas* strains via conjugation (3 h at 30 °C) on a 0.22 µm pore-size, 47 mm sterile filter (Millipore, UK), using LB as the medium. Transconjugants were selected on LB containing gentamicin (50 µg ml^−1^) and using chloramphenicol (10 µg ml^−1^) as the counter selection against *E*. *coli*. A single crossover transconjugant was grown overnight in LB and plated onto LB containing gentamicin and 10% (w/v) sucrose to select for double crossover mutants. Homologous recombination was confirmed by PCR and DNA sequencing.

### Complementation of various *Pseudomonas* mutants

All the plasmids and primers used for complementation are listed in Tables S2 and S3. Either *phoX* (BIRD-1), *glpQI* or *glpQII* and their 5′ upstream regions (~300 bp) were cloned into the broad host-range plasmid *pBBRMCS1*-*km* ^49^ using the HiFi DNA Assembly Kit. *pBBR1MCS*-*km* was linearised with *Kpn*I and *Xba*I. Plasmids were transformed into the respective mutants via electroporation (18 Kv cm^−1^, 200 A resistance, and 25 Ω capacitance). Transconjugants were selected on LB kanamycin (50 μg ml^−1^).

### Quantification of alkaline phosphatase activity

Alkaline phosphatase activity was recorded according to Lidbury *et al*.^20^. Briefly, a cell culture (n = 3) was incubated with 20 μL *para*-nitrophenyl phosphate (*p*NPP) (final conc. 4 mM) until colour development occurred. The reaction was stopped using 25 μL NaOH (2 mM) and incubated for 10 min. Cell debris and precipitants were removed via centrifugation (2 min, 8,000 × *g*) prior to spectrophotometry (optical density 405 nm). A standard curve for *para*-nitrophenol was generated using a range of known concentrations (0, 34.8, 69.5, 139.1, 278.2, 556.4, μg mL^−1^).

### Bioinformatic analysis of Pi-liberating genes/proteins

Pfam domains associated with various proteins of interest were used as the queries for ‘function searches’ against a number of MGs and MTs deposited in the Integrated Microbial Genomes database (IMG/JGI) (https://img.jgi.doe.gov/). A list of MGs and MTs used in this study are outlined in Table S1. The number of hits retrieved for each MG or MT dataset were normalised by adjusting the total number of base pairs to 1 Gbp. BLASTP (cut off values, e-20, min. similarity 40%) was also conducted on each dataset using various characterised GlpQ homologs. To assess the diversity of sequences assigned to GlpQ, manually curated databases were established by downloading all the protein sequences in the IMG/JGI database annotated as possessing the Pfam03009 domain. A subsequent BLASTP search was performed using a relatively relaxed stringency (e-10). After sequences were assigned to a protein from a specific strain, the higher taxonomic rank of that strain was determined using the NCBI taxonomy browser tool https://www.ncbi.nlm.nih.gov/Taxonomy/Browser/wwwtax.cgi).

## Electronic supplementary material

Supplementary information

Table S1
