# Supplementary material for: Identification of extracellular glycerophosphodiesterases in Pseudomonas and their role in soil organic phosphorus remineralisation
Source: Sci Rep. 2017 May 19;7:2179. doi: 10.1038/s41598-017-02327-6 (PMC5438359; doi:10.1038/s41598-017-02327-6)
Supplement: Supplementary file 1 — Supplementary information [file 41598_2017_2327_MOESM1_ESM.doc]

Identification of extracellular glycerophosphodiesterases in *Pseudomonas* and their role in soil organic phosphorus remineralisation

Ian D. E. A. Lidbury^1^, Andrew R. J. Murphy^1^, Tandra D. Fraser^3^, Gary D. Bending^1^, Alexandra M. E. Jones^1^, Jonathan D. Moore^2^, Andrew Goodall^3^, Mark Tibbett^3^, John P. Hammond^3, 4^, David J. Scanlan^1^, Elizabeth M. H. Wellington^1^

^1^ School of Life Sciences, University of Warwick, Gibbet Hill Road, Coventry, West Midlands, CV4 7AL, United Kingdom

^2^ The Earlham Institute, Norwich Research Park, Norwich, NR4 7UH, United Kingdom

^3^ School of Agriculture, Policy, and Development, University of Reading, Earley Gate, Whiteknights, Reading RG6 6AR, United Kingdom

^4^ Southern Cross Plant Science, Southern Cross University, Lismore NSW 2480, Australia

Corresponding author: Ian D. E. A. Lidbury^1^; email, [i.lidbury@warwick.ac.uk](mailto:i.lidbury@warwick.ac.uk); Tel. +44 (0) 24 765 75874; Fax +44 (0)24 7652 2052.

**Supplementary information**

**Supplementary Figures**

**Figure S1** Identification of the key residues required for glycerolphosphodiester phosphodiesterase activity in various bacterial strains. The multiple alignment was performed using MUSCLE and visualised using the web-based server BOXSHADE v3.21 (<http://www.ch.embnet.org/software/BOX_form.html>). The black shading represented >50% identity, the grey shading represents >50% similarity. The coloured boxes indicate the key conserved residues (histidine, aspartic acid, glutamic acid). IMG Gene IDs: GlpQII_S.pneumo, 641572439; GlpQ_S.pneumoni, 641571566; MPNA4200, *Mycoplasma pneumonia,* 2512008150; UgpQ_E.coli, 646934160; GlpQI_DSM4166, 651178041; glpQ, *Haemophilus influenzae* 2585946559; GlpQ_E.coli, 637002210; GlpQII_SBW25; 649638195; PA2352, *P. aeruginosa,* 637052751;YhdW_*B.subtilis*, 2637210117; GlpQ_B.cereus, 2586049640.

**Figure S2** Alkaline phosphatase activity determined by production of para-nitrophenol (*p*NP) from the cleavage of para-nitrophenol phosphate (*p*NPP) in *Pseudomonas stutzeri* DSM4166 strains. Cells were either grown in Pi-replete (Pi = 1 mM, black bars), or Pi-deplete (Pi = 50 M, grey bars). Cells were harvested during mid-exponential phase. Results presented are the mean of triplicate cultures. Error bars denote standard deviation.

**Figure S3** Alkaline phosphatase activity determined by production of para-nitrophenol (*p*NP) from the cleavage of para-nitrophenol phosphate (*p*NPP) in *Pseudomonas putida* BIRD-1 strains. Cells were either grown in Pi-replete (Pi = 1 mM, black bars), or Pi-deplete (Pi = 50 µM, grey bars). Cells were harvested during mid-exponential phase. Results presented are the mean of triplicate cultures. Error bars denote standard deviation.

**Figure S4 Specific** growth rates of various *Pseudmonas putida* BIRD-1 strains on differing phosphorus compounds. The wild type (Red), *ΔphoX* (blue) or *ΔphoBR* (green) was grown on orthophosphate, glycerol-3-phosphate (circles) or phosphocholine (diamonds) as the sole source of phosphorus (100 μM). Results presented are the mean of triplicate cultures. Error bars denote standard deviation.

**Figure S5** Co-cultivation of *Pseudomonas putida* BIRD-1 strains. Either the *phoX* mutant or the *phoBR* mutant was co-cultivated with the parental wild type strain. Minimal A medium was either supplemented with 100 μM phosphorylcholine (Pch) or 100 μM *sn*-glycerol-3-phosphate (G3P). Cultures were incubated overnight. Results presented are the mean of triplicate cultures. Error bars denote standard deviation.

**Figure S6** Co-cultivation of the BIRD-1 *phoBR* mutant with its parental wild type strain under conditions of either high Pi or low Pi. Colony forming units were recorded aftert 24 hours growth. Results are the mean of triplicate experiments. Error bars denote standard deviation.

**Figure S7** Phylogenetic distribution of GlpQ-like sequences retrieved from the metagenomes and metatranscriptomes samples at the Centre INRS-Institut Armand-Frappier, Laval, Canada. A protein database containing all sequences containing the Pfam domain 03009 (genomic DNA only) deposited in the IMG/JGI database was manually curated. The relative abundance at the phylum level **(A)** and class level **(B)**.

Figure S1


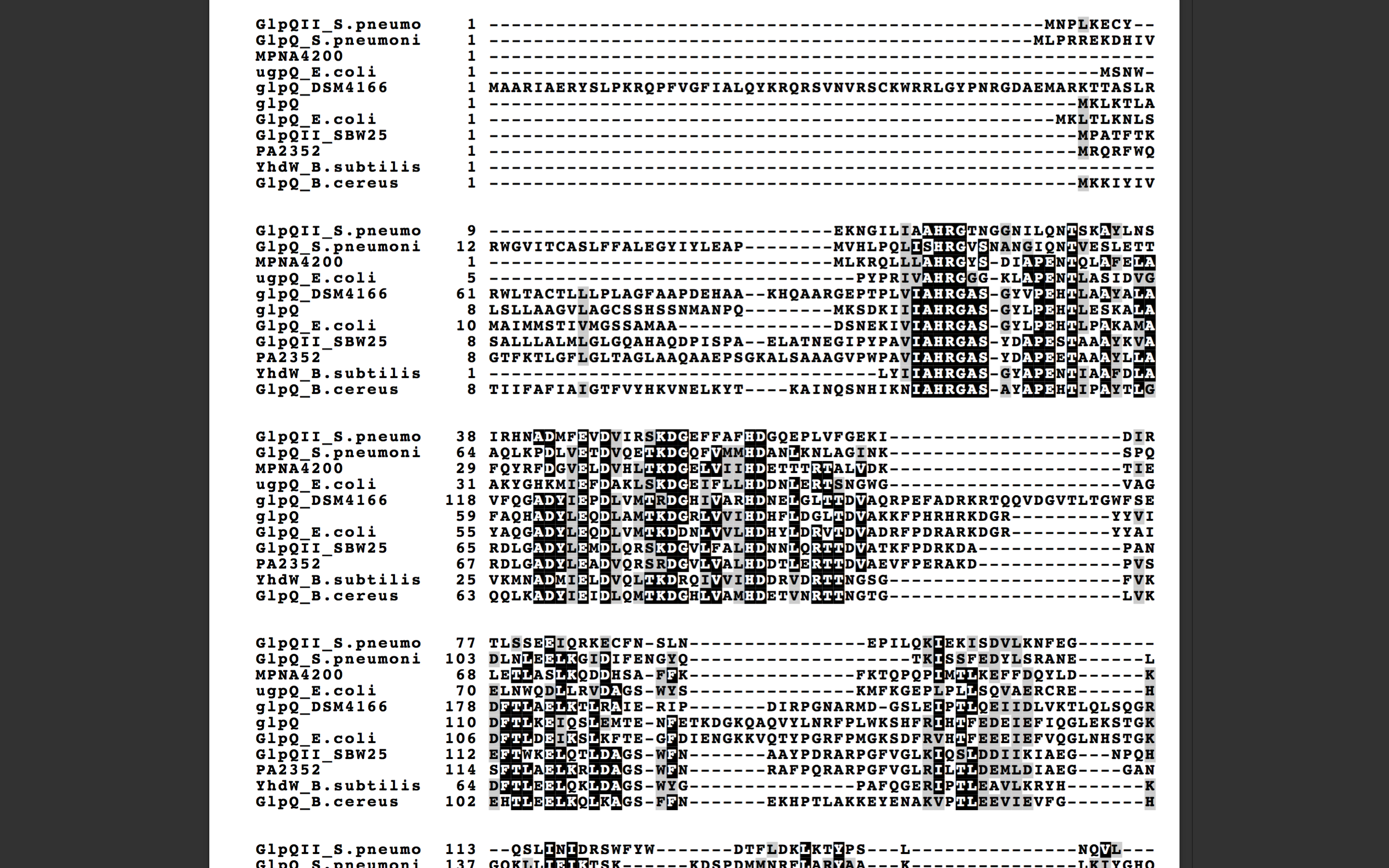


Figure S2


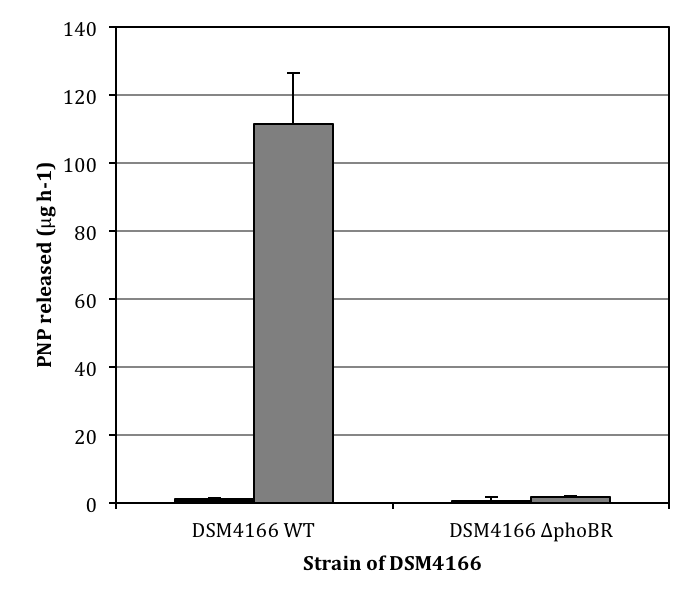


Figure S3


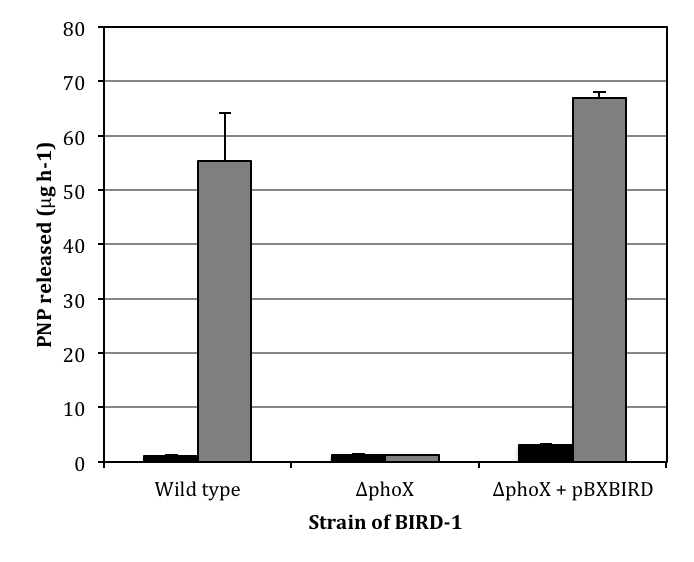


Figure S4


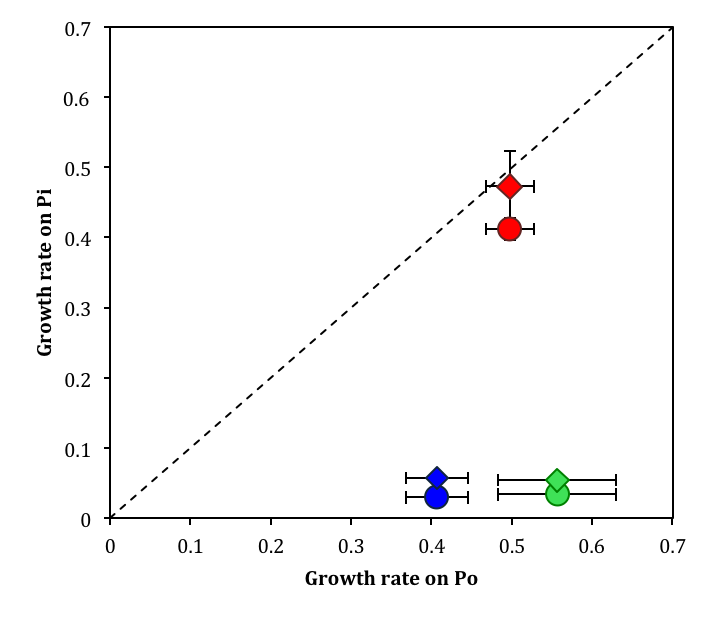


Figure S5


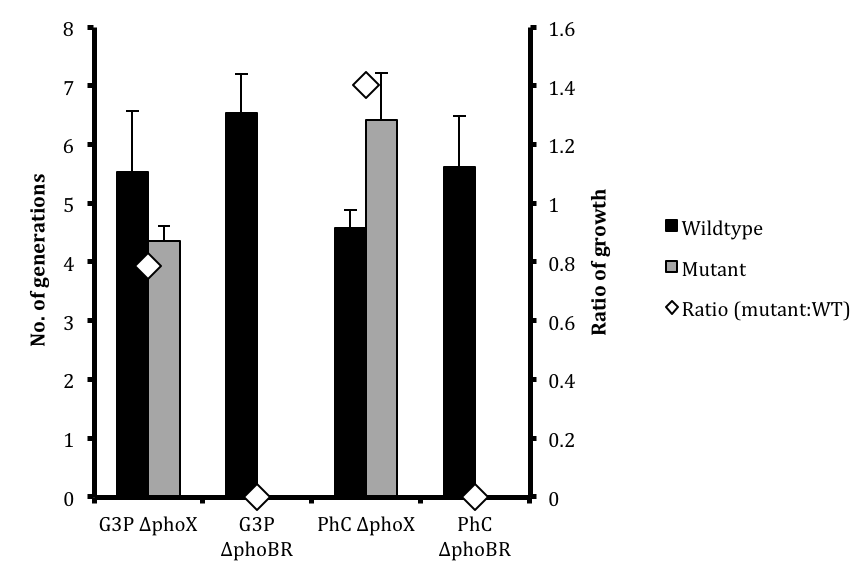


Figure S6


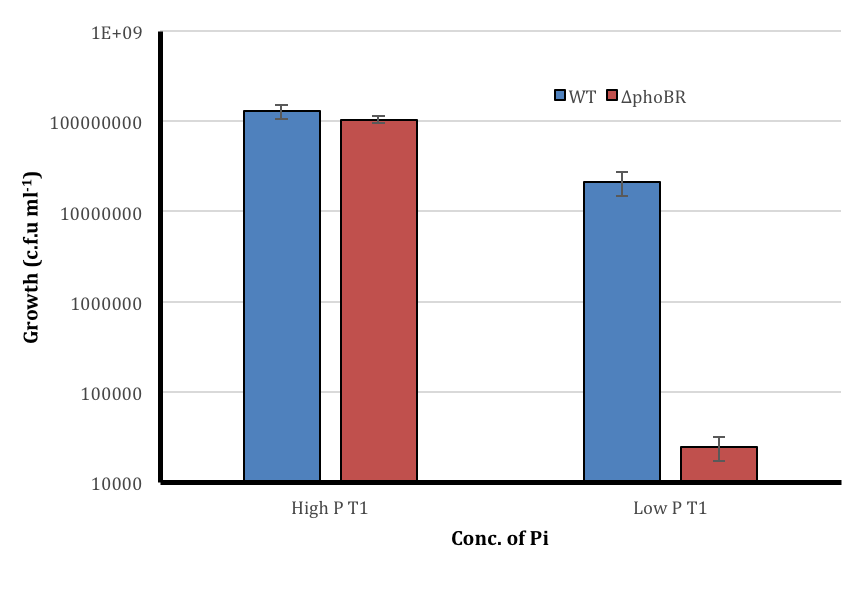


Figure S7


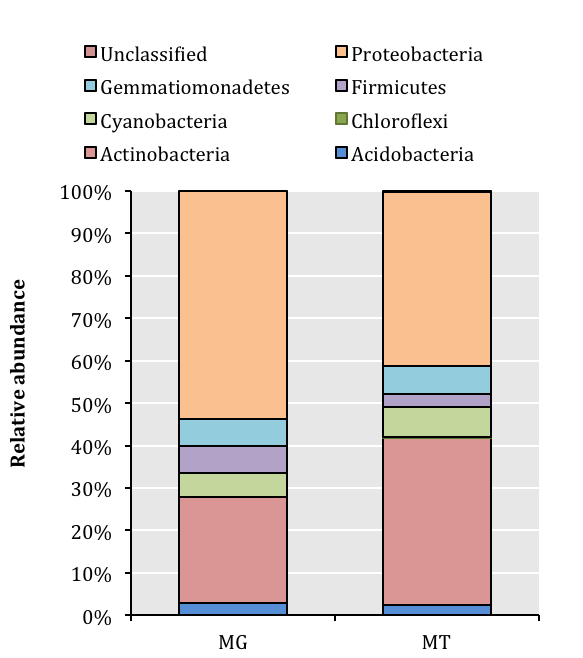

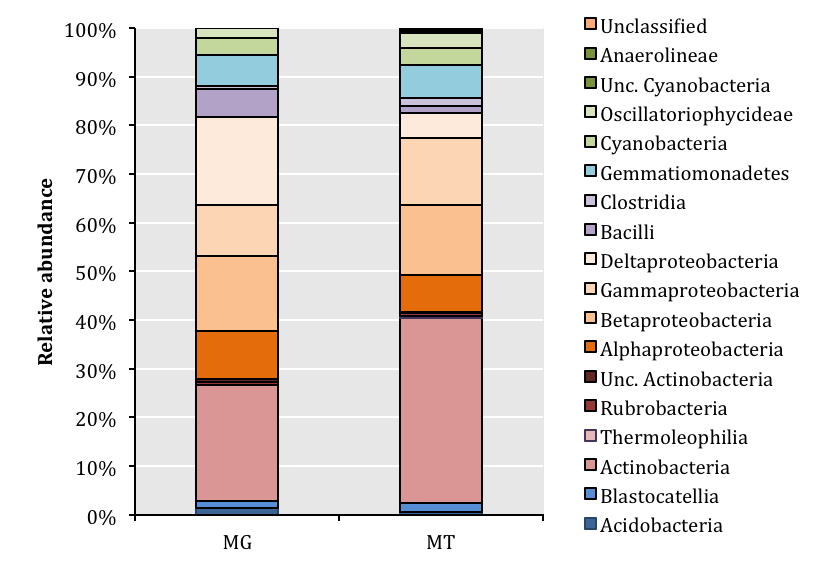


(A)

(B)

Table S1. Full list of metagenomes and metatranscriptomes used in this study deposited in the IMG/JGI database.

(See excel spreadsheet)

Table S2. A list of strains and plasmids used in this study

| **Strain/plasmids** | **Description** | **Reference** |
| --- | --- | --- |
| *Pseudomonas putida* BIRD-1 | Wild type strain |  |
| *Pseudomonas stutzeri* DSM4166 | Wild type strain | Lu et al. 2013 |
| *Pseudomonas fluorescens* SBW25 | Wild type strain | 2001 |
| *E. coli* S17.1 | Electrocompetent cells used for mating | Simon et al. 1983 |
| *E. coli* JM109 | Routine host for cloning | Sambrook et al. 2001 |
| BIRD-1_*phoX*­*::Gm* | Wild type BIRD-1 with disrupted *phoX* | This study |
| BIRD-1_*phoX*­*::Gm* + *phoX:BIRD-1* | *phoX* mutant complemented with pILAM001 | This study |
| BIRD-1_*phoXBR::Gm* | Wild type BIRD-1 with disrupted *phoBR* | This study |
| DSM4166_*glpQ::Gm* | Wild type DSM4166 with disrupted *glpQ* | This study |
| DSM4166_*phoBR::Gm* | Wild type DSM4166 with disrupted *phoBR* | This study |
| SBW25_*glpQII::Gm* | Wild type SBW25 with disrupted *glpQ* | This study |
| p34S-Gm | Source of the gentamicin cassette | Dennis and Zylstra, 1998 |
| pk18mobsacB | Suicide vector for *Pseudomonas*, KanR | Schäfer et al. 1994 |
| pBBR1MCS-km | Broad-host-range plasmid, KanR | Kovach et al. 1995 |
| pILAM001 | PPUBIRD1_1093 (*phoX*) and its promoter cloned into pBBR1MCS-km | This study |
| pBR:GlpQI  pBR:GlpQII | PSTAA_4169 (*glpQI*) and its promoter cloned into pBBR1MCS-km  PFLU_4789 (*glpQII*) and its promoter cloned into pBBR1MCS-km | This study  This study |

Table S3. A full list of primers used in this study

| **Primer** | **Sequence 5’ – 3’** | **Description** |
| --- | --- | --- |
| BIRD-1_*phoX*­_AF | attcgagctcggtacccgggGCCATCCTATCCACGTCCAT | Cloning region A (Upstream) of PPUBIRD1_1093 |
| BIRD-1_*phoX*­_AR | tagagtcgacGTTGCTGTTCAGTGGCGTAC | Cloning region A (Upstream) of PPUBIRD1_1093 |
| BIRD-1_*phoX*­_GF | gaacagcaacGTCGACTCTAGAGGATCCCCGGG | Amplification of gentamicin cassette – p34S-Gm |
| BIRD-1_*phoX*­_GR | agtcggtcaaTTGGCCGCGGCGTTGTGACA | Amplification of gentamicin cassette – p34S-Gm |
| BIRD-1_*phoX*­_BF | ccgcggccaaTTGACCGACTACACCAACGAC | Cloning region B (Downstream) of PPUBIRD1_1093 |
| BIRD-1_*phoX*­_AR | taaaacgacggccagtgccaGCAGCGAGTTCCAGGTGGA | Cloning region B (Downstream) of PPUBIRD1_1093 |
| BIRD-1_compl_F_Prom | CAATGGTACCGACGTACAGCAAGTCGAGC | Cloning the promoter of PPUBIRD1_1093 |
| BIRD-1_compl_R_Prom | CAATAAGCTTCAGGGTGTCCTTGTGTTCGC | Cloning the promoter of PPUBIRD1_1093 |
| BIRD-1_compl_F_PhoX | CATTAAGCTTATGAGTCGAGATACCGGCGA | Cloning 5’ end of PPUBIRD1_1093 |
| BIRD-1_compl_R_PhoX | CAATTCTAGAGTCAAACGGTGCCGATCTCAC | Cloning 3’ end of PPUBIRD1_1093 |
| DSM4166_*glpQ_*AF | attcgagctcggtacccgggCGATCCGTGTCAGGTAAAGC | Cloning region A (Upstream) of DSM4166_4169 |
| DSM4166_*glpQ_*AR | ctctagagtcgacATTTCGGCATCTCCTCGGTT | Cloning region A (Upstream) of DSM4166_4169 |
| DSM4166_*glpQ_*GF | gagatgccgaaatGTCGACTCTAGAGGATCCCCGGG | Amplification of gentamicin cassette – p34S-Gm |
| DSM4166_*glpQ_*GR | cttgaggttgtccTTGGCCGCGGCGTTGTGACA | Amplification of gentamicin cassette – p34S-Gm |
| DSM4166_*glpQ_*BF | acgccgcggccaaGGACAACCTCAAGACCCTCAG | Cloning region B (Downstream) of DSM4166_4169 |
| DSM4166_*glpQ_*BR | taaaacgacggccagtgccaTCAGCCAGATTCGGTCGAG | Cloning region B (Downstream) of DSM4166_4169 |
| DSM4166_*phoBR_*AF | tacgaattcgagctcggtacccgggCGCCTGGGAATACTGGAAGA | Cloning region A (Upstream) of DSM4166_ |
| DSM4166_*phoBR_*AR | ctctagagtcgacTCTTGTTGTCCTCTTCGCCTTT | Cloning region A (Upstream) of DSM4166_ |
| DSM4166_*phoBR_*GF | gaggacaacaagaGTCGACTCTAGAGGATCCCCGGG | Amplification of gentamicin cassette – p34S-Gm |
| DSM4166_*phoBR_*GR | taacccgcgatgaTTGGCCGCGGCGTTGTGACA | Amplification of gentamicin cassette – p34S-Gm |
| DSM4166_*phoBR_*BF | acgccgcggccaaTCATCGCGGGTTATCTGGAAAC | Cloning region B (Downstream) of DSM4166_ |
| DSM4166_*phoBR_*BR | cgttgtaaaacgacggccagtgccaACACCGGGAATGCTGGTGAT | Cloning region B (Downstream) of DSM4166_ |
| SBW25_*glpQ_*AF | atgattacgaattcgagctcggtacccgggCGTCTTCGCTGAAACGTAC | Cloning region A (Upstream) of PFLU_4796 |
| SBW25_*glpQ_*AR | tcctctagagtcgacTGCAGGTCCATTTCCAGG | Cloning region A (Upstream) of PFLU_4796 |
| SBW25_*glpQ_*GF | ggaaatggacctgcaGTCGACTCTAGAGGATCCCCGG | Amplification of gentamicin cassette – p34S-Gm |
| SBW25_*glpQ_*GR | tttcatcaccttgtcTTGGCCGCGGCGTTGTGA | Amplification of gentamicin cassette – p34S-Gm |
| SBW25_*glpQ_*BF | caacgccgcggccaaGACAAGGTGATGAAAGCCG | Cloning region B (Downstream) of PFLU_4796 |
| SBW25_*glpQ_*BR | cacgacgttgtaaaacgacggccagtgccaGTGTCGTTGATGGTGGCA | Cloning region B (Downstream) of PFLU_4796 |
| 4166_pBR:glpQIF | ccctcactaaagggaacaaaagctgggtacCCACCAGGTTGAGCCGCC | *glpQI* and the 350 bp upstream region |
| 4166_pBR:glpQIR | aattggagctccaccgcggtggcggccgctTCAGTCGGCGTTGCGCAG | *glpQI* and the 350 bp upstream region |
| SBW25_pBR:glpQIIF | ccctcactaaagggaacaaaagctgggtacCGGTTGAGGCCAACAAAC | *glpQII* and the 350 bp upstream region |
| SBW25_pBR:glpQIIR | aattggagctccaccgcggtggcggccgctTCAGTACTTATAGTCGTTCAACAG | *glpQII* and the 350 bp upstream region |

Table S4. Top hits (100) retrieved from BLASTP analysis against all *Betaproteobacteria* deposited in the National Centre for Biotechnology Information (NCBI) database using GlpQI from *Pseudmonas stutzeri* DSM4166 (PstA_4169) as the query.

| Identifier | % Identitiy | E value | Max Score |
| --- | --- | --- | --- |
| gi|810874157|ref|WP_046353414.1|;gi|984916345|gb|AMC33483.1| | 60.923 | 1.82E-134 | 396 |
| gi|991991110|ref|WP_060980218.1| | 60.377 | 4.96E-127 | 375 |
| gi|500125474|ref|WP_011801479.1|;gi|120593962|gb|ABM37401.1| | 57.231 | 6.51E-125 | 371 |
| gi|950256951|ref|WP_057293468.1|;gi|946117743|gb|KRB69957.1| | 57.846 | 3.66E-124 | 370 |
| gi|1082764368|gb|OGA82481.1| | 53.005 | 1.13E-122 | 367 |
| gi|930612485|ref|WP_054264604.1| | 51.604 | 2.35E-122 | 365 |
| gi|955159761|emb|CUI03813.1|;gi|962733609|emb|CUU27599.1| | 51.604 | 3.09E-122 | 365 |
| gi|948007617|ref|WP_056667075.1|;gi|945536354|gb|KQV91607.1| | 55.856 | 2.35E-120 | 360 |
| gi|1054572675|ref|WP_066341837.1| | 54.857 | 3.28E-120 | 359 |
| gi|916636177|ref|WP_051243268.1| | 52.909 | 3.63E-120 | 359 |
| gi|1054934326|ref|WP_066690363.1| | 55.891 | 5.54E-120 | 358 |
| gi|516490008|ref|WP_017878452.1| | 52.329 | 6.01E-120 | 359 |
| gi|1089400870|emb|SDY19097.1| | 52.707 | 6.84E-119 | 357 |
| gi|948083889|ref|WP_056742621.1|;gi|945965539|gb|KRA18615.1| | 54.955 | 1.95E-118 | 355 |
| gi|991998764|ref|WP_060986055.1| | 55.255 | 2.64E-118 | 355 |
| gi|764504295|ref|WP_044397183.1|;gi|763547088|gb|KJA10963.1| | 55.287 | 2.84E-118 | 355 |
| gi|1082857676|gb|OGB70820.1| | 55.193 | 2.87E-118 | 355 |
| gi|648281130|ref|WP_026062279.1| | 52.422 | 2.90E-118 | 356 |
| gi|738299421|ref|WP_036252522.1|;gi|672862941|gb|KFI05815.1| | 53.846 | 3.46E-118 | 355 |
| gi|1055398540|ref|WP_067061377.1| | 53.482 | 3.72E-118 | 355 |
| gi|1094982693|emb|SEL90772.1| | 51.467 | 4.24E-118 | 355 |
| gi|971868299|ref|WP_058937187.1| | 53.541 | 4.83E-118 | 355 |
| gi|1043083203|ref|WP_065344999.1|;gi|1042859591|gb|OBY87494.1| | 52.422 | 4.98E-118 | 355 |
| gi|971167592|gb|ALV05335.1| | 53.541 | 5.94E-118 | 355 |
| gi|736372322|ref|WP_034397478.1|;gi|673064860|gb|KFJ13554.1| | 52.137 | 7.86E-118 | 354 |
| gi|512594461|ref|WP_016454200.1|;gi|512036821|gb|EPD36644.1| | 52.422 | 9.58E-118 | 355 |
| gi|491909534|ref|WP_005665643.1|;gi|425720074|gb|EKU83000.1| | 52.841 | 1.16E-117 | 353 |
| gi|760100851|ref|WP_043783077.1| | 52.137 | 1.25E-117 | 353 |
| gi|504829293|ref|WP_015016395.1|;gi|407899244|gb|AFU48453.1| | 54.955 | 1.52E-117 | 353 |
| gi|950148387|ref|WP_057200533.1|;gi|946175287|gb|KRC27126.1| | 54.655 | 1.91E-117 | 353 |
| gi|1060095933|ref|WP_069104138.1|;gi|1059939381|gb|AOG21521.1| | 54.599 | 2.13E-117 | 353 |
| gi|1082762514|gb|OGA80641.1| | 54.303 | 2.35E-117 | 353 |
| gi|160367057|gb|ABX38670.1| | 52.137 | 2.44E-117 | 353 |
| gi|950180303|ref|WP_057227739.1|;gi|946295063|gb|KRD46326.1| | 54.006 | 2.46E-117 | 353 |
| gi|947507384|ref|WP_056171467.1|;gi|944766012|gb|KQO16971.1| | 54.303 | 2.74E-117 | 352 |
| gi|1063960560|gb|ODS72699.1| | 54.955 | 3.33E-117 | 352 |
| gi|503570726|ref|WP_013804802.1|;gi|333747711|gb|AEF92888.1| | 52.137 | 3.49E-117 | 352 |
| gi|950227144|ref|WP_057271236.1|;gi|946270652|gb|KRD22024.1| | 53.412 | 4.37E-117 | 352 |
| gi|493139489|ref|WP_006156398.1|;gi|373103388|gb|EHP44413.1| | 56.135 | 4.43E-117 | 352 |
| gi|512560258|ref|WP_016447242.1|;gi|512040450|gb|EPD40206.1| | 52.137 | 4.43E-117 | 353 |
| gi|1100220731|ref|WP_071361544.1|;gi|1098885837|gb|OIJ40567.1| | 52.557 | 5.18E-117 | 351 |
| gi|498150707|ref|WP_010464863.1| | 53.709 | 5.62E-117 | 352 |
| gi|738917709|ref|WP_036804532.1|;gi|669783222|emb|CDS49589.1| | 51.752 | 5.85E-117 | 351 |
| gi|496180091|ref|WP_008904598.1|;gi|363415940|gb|EHL23064.1| | 54.599 | 6.52E-117 | 351 |
| gi|495128138|ref|WP_007852949.1|;gi|394316551|gb|EJE53272.1| | 54.354 | 7.93E-117 | 351 |
| gi|1082745257|gb|OGA63975.1| | 54.006 | 9.34E-117 | 351 |
| gi|947982478|ref|WP_056642137.1|;gi|946082394|gb|KRB34924.1| | 54.655 | 1.04E-116 | 351 |
| gi|1082791413|gb|OGB08160.1|;gi|1082845983|gb|OGB59621.1| | 54.303 | 1.06E-116 | 351 |
| gi|827459207|ref|WP_047219261.1| | 52.941 | 1.18E-116 | 350 |
| gi|942697468|ref|WP_055402136.1|;gi|940790414|gb|KQB56237.1| | 54.006 | 1.57E-116 | 350 |
| gi|1074977440|ref|WP_070080760.1|;gi|1073964052|gb|AOV03772.1| | 52.149 | 2.07E-116 | 351 |
| gi|947390803|ref|WP_056056780.1|;gi|945577539|gb|KQW32528.1| | 53.709 | 2.20E-116 | 350 |
| gi|947748874|ref|WP_056410890.1|;gi|944775805|gb|KQO26698.1|;gi|944789672|gb|KQO40469.1|;gi|945182075|gb|KQS42611.1| | 53.709 | 2.63E-116 | 350 |
| gi|917069398|ref|WP_051676110.1| | 56.707 | 2.74E-116 | 349 |
| gi|950222622|ref|WP_057266945.1|;gi|946177256|gb|KRC29087.1| | 54.354 | 5.26E-116 | 349 |
| gi|759633525|ref|WP_043351239.1| | 55.521 | 6.47E-116 | 349 |
| gi|612098461|gb|EZP56846.1| | 53.079 | 6.63E-116 | 349 |
| gi|760139405|ref|WP_043820671.1| | 53.079 | 9.08E-116 | 350 |
| gi|752315362|gb|AJG17970.1| | 55.521 | 1.09E-115 | 349 |
| gi|1055037023|ref|WP_066787848.1| | 54.683 | 1.46E-115 | 348 |
| gi|1023915856|ref|WP_063461453.1|;gi|1023217869|gb|KZT14948.1| | 54.006 | 2.52E-115 | 348 |
| gi|920600186|ref|WP_053014057.1| | 54.085 | 2.69E-115 | 347 |
| gi|826173310|gb|AKJ31540.1| | 54.062 | 3.13E-115 | 348 |
| gi|1063947203|gb|ODS60019.1| | 53.709 | 1.00E-114 | 346 |
| gi|929042435|ref|WP_054065439.1| | 49.459 | 2.66E-114 | 345 |
| gi|515792438|ref|WP_017224822.1| | 53.681 | 3.15E-114 | 343 |
| gi|829803150|ref|WP_047349288.1|;gi|829487605|gb|KLR59504.1| | 53.295 | 6.28E-114 | 344 |
| gi|771666473|ref|WP_045237858.1| | 53.681 | 9.09E-114 | 344 |
| gi|770107626|gb|KJK23935.1| | 53.681 | 1.03E-113 | 343 |
| gi|971070315|ref|WP_058880544.1|;gi|970586141|gb|KUF38440.1| | 49.189 | 3.42E-113 | 342 |
| gi|1004406637|ref|WP_061540808.1|;gi|1004067138|gb|AMO96164.1| | 54.079 | 3.64E-113 | 342 |
| gi|927581105|ref|WP_053822389.1|;gi|927307005|gb|ALD91380.1| | 53.709 | 3.93E-113 | 342 |
| gi|1054774569|ref|WP_066538312.1| | 52.553 | 4.78E-113 | 342 |
| gi|946913617|ref|WP_055836250.1|;gi|944627873|gb|KQM79591.1| | 53.709 | 1.14E-112 | 340 |
| gi|754104231|ref|WP_041742101.1| | 53.776 | 1.24E-112 | 340 |
| gi|835354645|ref|WP_047471833.1| | 52.744 | 2.09E-112 | 338 |
| gi|1087351170|emb|SDC45128.1| | 52.395 | 2.10E-112 | 340 |
| gi|984943523|ref|WP_060784290.1|;gi|984759119|gb|KWW36071.1| | 50 | 2.12E-112 | 340 |
| gi|516084184|ref|WP_017514767.1|;gi|982559363|gb|KWR83570.1| | 49.735 | 2.41E-112 | 340 |
| gi|340553253|gb|AEK62628.1| | 53.776 | 2.46E-112 | 340 |
| gi|1010996658|ref|WP_061954776.1| | 52.568 | 5.34E-112 | 339 |
| gi|1064111790|gb|ODU11820.1|;gi|1064219530|gb|ODV14818.1| | 51.497 | 1.21E-111 | 339 |
| gi|950505372|ref|WP_057392168.1| | 50.409 | 1.23E-111 | 338 |
| gi|737915992|ref|WP_035881379.1| | 51.36 | 1.78E-111 | 338 |
| gi|499615940|ref|WP_011296674.1|;gi|72117604|gb|AAZ59867.1| | 51.94 | 2.14E-111 | 337 |
| gi|917765724|ref|WP_052279728.1|;gi|982563113|gb|KWR87320.1| | 51.479 | 2.77E-111 | 338 |
| gi|499834566|ref|WP_011515300.1|;gi|93353222|gb|ABF07311.1| | 48.148 | 4.53E-111 | 337 |
| gi|981063972|ref|WP_059410513.1| | 53.172 | 5.86E-111 | 337 |
| gi|1035740291|ref|WP_064576461.1| | 53.614 | 6.63E-111 | 336 |
| gi|1028879012|ref|WP_064045740.1|;gi|1028445998|gb|OAI73802.1| | 50.136 | 7.00E-111 | 336 |
| gi|1021305945|ref|WP_063328074.1|;gi|1020000224|gb|KZK30943.1| | 52.553 | 9.70E-111 | 336 |
| gi|763064650|ref|WP_043946402.1| | 50.136 | 1.10E-110 | 335 |
| gi|489362687|ref|WP_003269583.1|;gi|378965273|emb|CCF97162.1| | 49.864 | 1.55E-110 | 335 |
| gi|495921446|ref|WP_008646025.1|;gi|429500367|gb|EKZ98743.1| | 51.057 | 1.94E-110 | 335 |
| gi|502971979|ref|WP_013206955.1|;gi|299072803|emb|CBJ44158.1| | 49.864 | 3.65E-110 | 334 |
| gi|737883434|ref|WP_035849942.1| | 51.479 | 3.80E-110 | 335 |
| gi|656002203|ref|WP_029043655.1| | 52.568 | 1.05E-109 | 333 |
| gi|518276839|ref|WP_019447047.1| | 51.662 | 1.16E-109 | 333 |
| gi|1054722399|ref|WP_066487913.1| | 53.823 | 1.26E-109 | 331 |
| gi|752557057|ref|WP_041228289.1| | 51.796 | 1.36E-109 | 333 |

Table S5. List of the genomes extracted from the IMG/JGI database used to determine the distribution of Pfam03009-containing enzymes.

| IMG Genome ID | Genome Name / Sample Name | Status | Genome Size * assembled | Gene Count * assembled |
| --- | --- | --- | --- | --- |
| 637000016 | *Bacillus cereus* ATCC 10987 | Finished | 5432652 | 6126 |
| 637000017 | *Bacillus cereus* ATCC 14579 | Finished | 5427083 | 5513 |
| 2630968303 | *Bacillus cereus* D17 | Finished | 5590358 | 5870 |
| 2636415870 | *Bacillus subtilis* 916 | Finished | 3981674 | 3931 |
| 2630968642 | *Bacillus subtilis* ATCC 19217 | Finished | 3959897 | 3838 |
| 2663762614 | *Bacillus subtilis* B4071 | Permanent Draft | 4124910 | 4470 |
| 2645728053 | *Bacillus subtilis* BS49 | Finished | 4251652 | 4405 |
| 646311909 | *Bacillus subtilis subtilis* 168 | Finished | 4215606 | 4354 |
| 651053020 | *Escherichia coli* DH1 (ME8569) | Finished | 4621430 | 4409 |
| 2630968533 | *Escherichia coli* ECONIH1 | Finished | 5559642 | 5494 |
| 2558309052 | *Escherichia coli* JJ1886 | Finished | 5308284 | 5213 |
| 646311926 | *Escherichia coli* K-12, MG1655 | Finished | 4639675 | 4497 |
| 651053022 | *Escherichia coli* NA114 | Finished | 4935241 | 4944 |
| 637000107 | *Escherichia coli* O157:H7 EDL933 (EHEC) | Finished | 5620522 | 5633 |
| 649633050 | *Haemophilus influenzae* F3031 | Finished | 1985832 | 1850 |
| 2627853926 | *Haemophilus influenzae* Hi375 | Finished | 1850897 | 1829 |
| 2554235457 | *Haemophilus influenzae* KR494 (unscreened) | Finished | 1856176 | 1820 |
| 637000125 | *Haemophilus influenzae* NTHi 86-028NP | Finished | 1913428 | 1913 |
| 650377946 | *Haemophilus influenzae* R2866 | Finished | 1932306 | 1875 |
| 2551306331 | *Mycoplasma pneumoniae* 19294 | Finished | 781002 | 798 |
| 2511231209 | *Mycoplasma pneumoniae* 309 | Finished | 817176 | 749 |
| 2667527723 | *Mycoplasma pneumoniae* 4358 | Permanent Draft | 817496 | 823 |
| 648231715 | *Mycoplasma pneumoniae* FH | Finished | 811088 | 670 |
| 2565956577 | *Pseudomonas aeruginosa* LESlike5 | Finished | 6543350 | 6077 |
| 2627853962 | *Pseudomonas aeruginosa* NCGM1900 | Finished | 6814936 | 6456 |
| 2667527464 | *Pseudomonas aeruginosa* NCTC10332 | Finished | 6316979 | 5922 |
| 2558309066 | *Pseudomonas aeruginosa* PA1 (unscreened) | Finished | 6528877 | 5876 |
| 2636416113 | *Pseudomonas aeruginosa* PAO1 | Permanent Draft | 6058177 | 6401 |
| 637000218 | *Pseudomonas aeruginosa* PAO1 | Finished | 6264404 | 5671 |
| 2597490176 | *Pseudomonas alkylphenolia* KL28 | Finished | 5764622 | 5426 |
| 2648501814 | *Pseudomonas balearica* DSM 6083 | Finished | 4383480 | 4178 |
| 650716074 | *Pseudomonas brassicacearum* *brassicacearum* NFM421 | Finished | 6843248 | 6176 |
| 2585427645 | *Pseudomonas chlororaphis* PA23 | Finished | 7122173 | 6286 |
| 2540341139 | *Pseudomonas denitrificans* ATCC 13867 | Finished | 5696307 | 5135 |
| 637000219 | *Pseudomonas entomophila* L48 | Finished | 5888780 | 5293 |
| 2561511156 | *Pseudomonas fluorescens* A506 | Finished | 6019547 | 5493 |
| 2554235341 | *Pseudomonas fluorescens* CHA0 | Finished | 6867980 | 6199 |
| 2511231156 | *Pseudomonas fluorescens* F113 | Finished | 6845832 | 5952 |
| 2648501162 | *Pseudomonas fluorescens* PCL1751 | Finished | 6143950 | 5747 |
| 637000221 | *Pseudomonas fluorescens* Pf0-1 | Finished | 6438405 | 5857 |
| 2503538034 | *Pseudomonas fluorescens* R124 | Finished | 6299732 | 5572 |
| 649633086 | *Pseudomonas fluorescens* SBW25 | Finished | 7147633 | 6492 |
| 2585427659 | *Pseudomonas fluorescens* UK4 | Finished | 6064456 | 5299 |
| 2505679082 | *Pseudomonas fulva* 12-X | Finished | 4920769 | 4574 |
| 650716075 | *Pseudomonas mendocina* NK-01 | Finished | 5434353 | 5035 |
| 2516653025 | *Pseudomonas monteilii* SB3078 (v1.0) | Finished | 5866715 | 5525 |
| 637000220 | *Pseudomonas protegens* Pf-5 | Finished | 7074893 | 6257 |
| 650377963 | *Pseudomonas putida* BIRD-1 | Finished | 5731541 | 5046 |
| 2579778942 | *Pseudomonas putida* DLL-E4 | Finished | 6484062 | 5946 |
| 640427132 | *Pseudomonas putida* F1 | Finished | 5959964 | 5423 |
| 641522645 | *Pseudomonas putida* GB-1 | Finished | 6078430 | 5515 |
| 2563366586 | *Pseudomonas putida* H8234 | Finished | 6870827 | 6482 |
| 637000222 | *Pseudomonas putida* KT2440 | Finished | 6181863 | 5481 |
| 2554235365 | *Pseudomonas putida* NBRC 14164 | Finished | 6156701 | 5544 |
| 641522646 | *Pseudomonas putida* W619 | Finished | 5774330 | 5292 |
| 2517572175 | P*seudomonas* sp. FGI182 | Finished | 5891312 | 5365 |
| 2636415557 | *Pseudomonas* sp. StFLB209 | Finished | 6332373 | 5709 |
| 2558309084 | *Pseudomonas* sp. TKP | Finished | 7012672 | 6413 |
| 2519899536 | *Pseudomonas* sp. UW4 | Finished | 6183388 | 5517 |
| 2558309062 | *Pseudomonas* sp. VLB120 | Finished | 5966222 | 5580 |
| 2574179801 | *Pseudomonas stutzeri* 19SMN4 | Finished | 4833395 | 4485 |
| 2565956579 | *Pseudomonas stutzeri* 28a24 | Finished | 4731359 | 4286 |
| 640427133 | *Pseudomonas stutzeri* A1501 | Finished | 4567418 | 4237 |
| 2518645567 | *Pseudomonas stutzeri* AN10, CCUG 29243 | Finished | 4709064 | 4374 |
| 651053060 | *Pseudomonas stutzeri* CMT.A.9, DSM 4166 | Finished | 4689946 | 4374 |
| 2519899778 | *Pseudomonas stutzeri* JM3000, DSM 10701 | Finished | 4174118 | 3888 |
| 650716077 | *Pseudomonas stutzeri* Lautrop AB 201, ATCC 17588 | Finished | 4547930 | 4287 |
| 2507149015 | *Pseudomonas stutzeri* RCH2 | Finished | 4600489 | 4412 |
| 2506783024 | *Pseudomonas syringae* CC1557 (CC1557) | Finished | 5714345 | 5792 |
| 2597489907 | *Pseudomonas syringae* pv. actinidia ICMP 9617 | Finished | 6497695 | 5462 |
| 637000223 | *Pseudomonas syringae* pv. phaseolicola 1448A | Finished | 6112448 | 5436 |
| 2645727672 | *Pseudomonas syringae* pv. syringae B301D | Finished | 6094819 | 5375 |
| 2523533564 | *Pseudomonas syringae* pv. syringae B64 | Finished | 5930035 | 5021 |
| 637000224 | *Pseudomonas syringae* pv. syringae B728a | Finished | 6093698 | 5245 |
| 2639763113 | *Pseudomonas syringae* pv. syringae HS191 | Finished | 5950211 | 5234 |
| 2508501074 | *Pseudomonas syringae* pv. tomato DC3000 (DC3000 gold standard) | Finished | 6538260 | 5955 |
| 2540341132 | *Streptococcus pneumoniae* gamPNI0373 | Finished | 2064154 | 2225 |
| 643692041 | *Streptococcus pneumoniae* JJA | Finished | 2120234 | 2193 |
| 641522652 | *Streptococcus pneumoniae* sv. 19A-6 Hungary | Finished | 2245615 | 2222 |
| 643692043 | *Streptococcus pneumoniae* sv. 19F Taiwan19F-14 | Finished | 2112148 | 2114 |
| 648028055 | *Streptococcus pneumoniae* sv. 6B 670-6B | Finished | 2240045 | 2416 |
| 648028057 | *Streptococcus pneumoniae* TCH8431/19A | Finished | 2088772 | 2354 |
